# Eosinophil Deficiency Promotes Aberrant Repair and Adverse Remodeling Following Acute Myocardial Infarction

**DOI:** 10.1016/j.jacbts.2020.05.005

**Published:** 2020-07-08

**Authors:** Iqbal S. Toor, Dominik Rückerl, Iris Mair, Rob Ainsworth, Marco Meloni, Ana-Mishel Spiroski, Cecile Benezech, Jennifer M. Felton, Adrian Thomson, Andrea Caporali, Thomas Keeble, Kare H. Tang, Adriano G. Rossi, David E. Newby, Judith E. Allen, Gillian A. Gray

**Affiliations:** a British Heart Foundation/University Centre for Cardiovascular Science, Queen's Medical Research Institute, University of Edinburgh, Edinburgh, United Kingdom; b Faculty of Biology, Medicine and Health, School of Biological Sciences, University of Manchester, Manchester, United Kingdom; c MRC Centre for Inflammation Research, Queen's Medical Research Institute, University of Edinburgh, Edinburgh, United Kingdom; d Division of Pathology, Deanery of Molecular, Genetic and Population Sciences, University of Edinburgh, Edinburgh, United Kingdom; e Essex Cardiothoracic Centre, Basildon and Thurrock Hospitals NHS Foundation Trust, Essex, United Kingdom; f School of Medicine, Anglia Ruskin University, Cambridge, United Kingdom

**Keywords:** eosinophil, remodeling, STEMI, BMD, bone marrow derived, IL, interleukin, MI, myocardial infarction, mRNA, messenger RNA, PBS, phosphate-buffered saline, qPCR, quantitative polymerase chain reaction, STEMI, ST-segment elevation myocardial infarction, WT, wild-type

## Abstract

In ST-segment elevation myocardial infarction of both patients and mice, there was a decline in blood eosinophil count, with activated eosinophils recruited to the infarct zone. Eosinophil deficiency resulted in attenuated anti-inflammatory macrophage polarization, enhanced myocardial inflammation, increased scar size, and deterioration of myocardial structure and function. Adverse cardiac remodeling in the setting of eosinophil deficiency was prevented by interleukin-4 therapy.

## Highlights

• A drop in eosinophil blood count is associated with recruitment of eosinophils to the heart during repair following clinical and experimental MI.
• Genetic and pharmacological eosinophil depletion leads to increased adverse remodeling in experimental MI.
• Eosinophils are required for acquisition of an anti-inflammatory macrophage phenotype, a shift to resolution of inflammation and mature scar formation during infarct repair.
• IL-4 therapy is able to rescue the adverse remodeling phenotype in conditions of eosinophil deficiency.

## Visual Abstract

**Figure:**
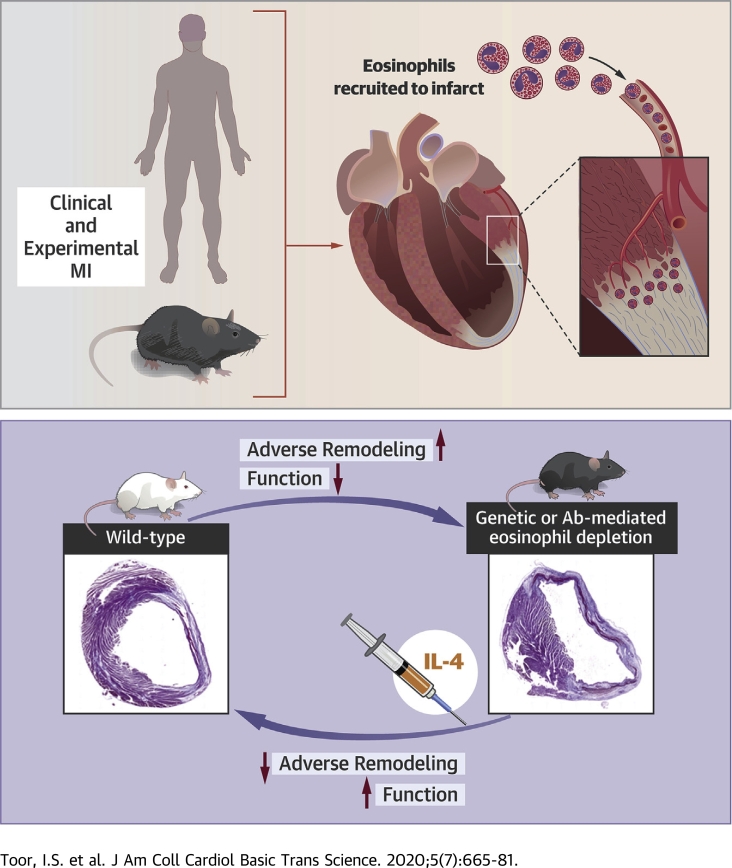

Myocardial infarction (MI) occurs most commonly following acute thrombotic occlusion of a coronary artery, and triggers an acute inflammatory response. Within hours, neutrophils are recruited to the infarcted myocardium followed by infiltration of proinflammatory monocytes (1). Acquisition of a proresolution proliferative macrophage phenotype is critical to successful infarct repair (2). Failure to expand the number of CD206^+^ proresolution macrophages following MI is associated with increased cardiac rupture and adverse cardiac remodeling because of disrupted collagen fibril formation during infarct healing (2). Interventions that polarize macrophages toward this phenotype, including interleukin (IL)-4 (2,3), improve infarct healing, but the endogenous mechanisms that regulate repair are not well understood.

Macrophage phenotype can be determined by environmental factors and importantly by cells of the innate and adaptive immune system, including eosinophils. Tissue eosinophilia is commonly associated with helminth infection, allergy, and gastrointestinal disorders. In these settings, eosinophils are effector cells with proinflammatory and destructive capabilities through the secretion of cytotoxic granules, cationic proteins, and proteolytic enzymes (4). However, eosinophils also express a number of immunomodulatory cytokines and lipid mediators implicated in the resolution of inflammation (4). Previous studies have linked peripheral blood eosinophil count MI to short-term risk of mortality in low- to intermediate-risk patients (5) and high-risk patients (6,7) following myocardial infarction. However, whether eosinophils have any role in repair of the adult mammalian heart is so far unknown.

The primary goal of the present study was to address the role of eosinophils in repair of the heart following MI in the setting of patients with acute ST-segment elevation MI (STEMI) and in an experimental model of MI in mice with genetic (ΔdblGATA) (8) or pharmacologic depletion of eosinophils.

## Methods

### Patient selection with STEMI

All patients included in the STEMI registry were ≥18 years of age, had chest pain of <12 h duration, and had persistent ST-segment elevation in at least 2 contiguous leads of the electrocardiogram (9). Patients with a history of stable angina undergoing elective percutaneous coronary intervention were used as the control group. This study was reviewed and approved by the Essex Cardiothoracic Centre Clinical Governance body.

### Animal experiments

Twelve- to 14-week-old wild-type (WT) male BALB/c mice and C57BL/6 mice were purchased from Harlan Laboratories (Huntingdon, United Kingdom). ΔdblGATA mice on a BALB/c background (8,10) were bred and maintained at the University of Edinburgh. All animal experiments were approved by the University of Edinburgh Animal Welfare and Ethical Review Board and the UK Home Office.

### Infarct model

MI was induced by permanent ligation of the left coronary artery in isoflurane anesthetized mice as previously described (11). Twenty-four hours after surgery, a tail blood sample was collected for assay of troponin I (Life Diagnostics High Sensitivity Mouse Cardiac Troponin-I ELISA kit, Life Diagnostics, West Chester, Pennsylvania) to assess the extent of myocardial injury.

### Anti-Siglec-F antiserum depletion of eosinophils

In the eosinophil depletion study, C57BL/6 mice received 100 μl of either sheep anti-Siglec-F polyclonal antiserum or sheep pre-immune serum (both gifted by Professor Paul Crocker, University of Dundee, Dundee, United Kingdom) via intraperitoneal injection at 1 day before and 3 days after MI.

### Rescue experiments in ΔdblGATA mice

Bone marrow–derived (BMD) eosinophils were adoptively transferred by intraperitoneal injection into ΔdblGATA mice at 1 day before and 3 days after MI. Purity of >90% for eosinophils was confirmed prior to intraperitoneal injection by flow cytometric analysis.

For IL-4 replenishment, 5 μg of recombinant murine IL-4 (PeproTech, Rocky Hill, New Jersey) complexed to 25-μg anti-IL-4 antibody (clone 11B11, Bio X Cell, Lebanon, New Hampshire) in 100-μl sterile phosphate-buffered saline (PBS) or 100-μl sterile PBS was administered to ΔdblGATA mice via intraperitoneal injection at day 1 post-MI.

### Cardiac imaging

Left ventricular structure and function was assessed using high-resolution ultrasound (VisualSonics Vevo 770, VisualSonics, Toronto, Canada), under light isoflurane anesthesia, ensuring that the heart rate was maintained >450 beats/min.

### Histopathology

Eosinophils were detected in samples of paraffin-embedded infarcted human myocardium, obtained from the Edinburgh Brain and Tissue Bank (Supplemental Table 1), using eosinophil peroxidase antibody (supplied by Elizabeth Jacobsen, Mayo Clinic, Scottsdale, Arizona).

Paraffin embedded mouse heart sections were stained with Masson’s trichrome (infarct size) and picrosirius red (polarized light to collagen fiber status using polarized light). Angiogenesis was determined by detection of CD31 immunopositive vessels (rabbit anti-mouse CD31, 1:50, Abcam, Cambridge, United Kingdom).

### Transmission electron microscopy

Mouse hearts were fixed in 1% osmium tetroxide in 0.1-M sodium cacodylate X3 and embedded in TAAB 812 resin (TAAB Laboratories Equipment, Reading, United Kingdom). Ultrathin sections, 60-nm thick, were stained in uranyl acetate and lead citrate and then viewed in a JEOL JEM-1400 Plus transmission electron microscope (JEOL, Tokyo, Japan). Eosinophils were identified by their typical morphology with crystalloid granules.

### Flow cytometry

Immunofluorescence staining was performed on tail blood samples and single-cell suspensions from heart digests, pericardial adipose, spleens, and peritoneal cells. Flow cytometric analysis was performed on an LSR II instrument (BD Biosciences, Franklin Lakes, New Jersey) and analyzed using FlowJo software v9.9.4 (Tree Star, Ashland, Oregon). Infarct zone CD45^+^CD11b^+^Ly6G^-^F4/80^+^ macrophages were sorted by FACS using a FACS ARIA II flow cytometer (BD Biosciences). Results for the heart digests are expressed as cell number per infarct zone or remote zone, and total counts were calculated for spleens and the peripheral blood.

### RNA extraction and real-time quantitative polymerase chain reaction

RNA was extracted from infarct zone tissue (RNeasy Mini Kit, Qiagen, Hilden, Germany) and reverse transcribed to complementary DNA with the QuantiTect Reverse Transcription Kit (Qiagen). Real-time quantitative polymerase chain reaction (qPCR) was performed using TAQman gene expression assays (Thermo Fisher Scientific, Waltham, Massachusetts) (Supplemental Table 2). Messenger RNA (mRNA) expression levels were normalized for Rpl32 (housekeeping gene) expression and are presented as fold changes. Genes were selected based on prior microarray data from the lab (G.A. Gray, unpublished data, February 2016) that identified them as being significantly modified during the early stages of scar formation, between 3 and 7 days following MI.

### Tissue cytokine assay

Left ventricular infarct zone tissue was collected 4 days after induction of MI and snap frozen. Tissue cytokines in the heart protein extracts were assayed using the mouse Th2 Panel LEGENDplex assay (BioLegend, San Diego, California), according to the manufacturer’s instructions.

### Statistics

Continuous variables are expressed as mean ± SEM, unless otherwise stated. The Kolmogorov-Smirnov test was used to test for the normality of data. Continuous variables with a normal distribution were analyzed using Student's *t*-test, or by analysis of variance. Differences in blood eosinophil and neutrophil counts between stable angina and STEMI patients after MI were compared by 1-way analysis of variance, and multiple comparison was taken into account using a modified least-squares difference (Bonferroni) test. Variables with a nonparametric distribution were analyzed using Wilcoxon rank sum test or Wilcoxon signed rank test for multiple comparison. Categorical data are presented as counts and as a percentage. Chi-square tests was used for comparisons of categorical variables. A p value <0.05 was considered statistically significant. All statistical analyses were performed with SPSS 21.0 statistical software package (IBM Corp., Armonk, New York).

## Results

### Eosinophils are depleted from the blood and accumulate in the myocardium of patients after acute MI

**Figure 1:**
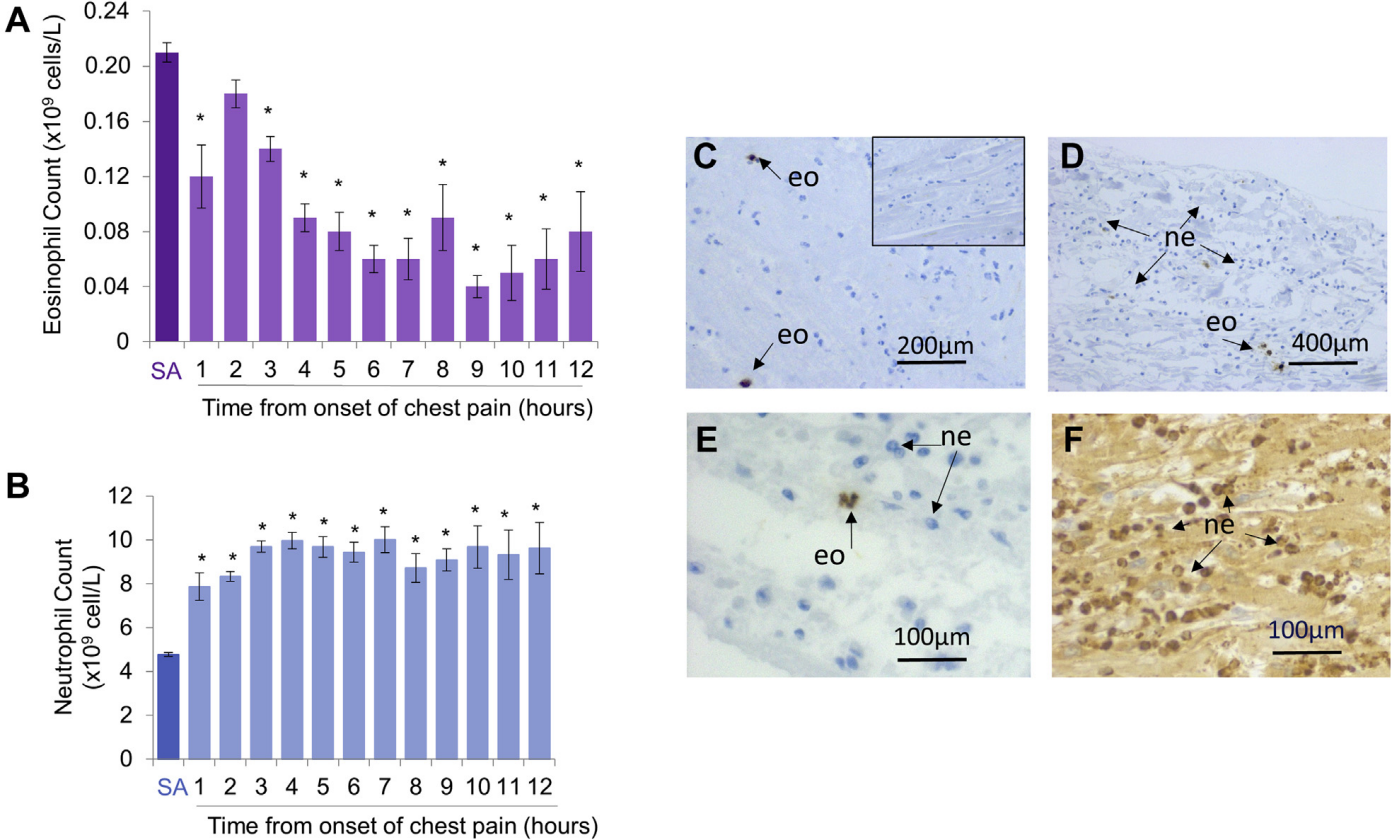
Blood Eosinophil Count Is Reduced, Independently of Changes in Neutrophil Count in ST-Segment Elevation Myocardial Infarction Patients, While Eosinophils Accumulate in the Human Heart Following MI **(A)** Peripheral blood eosinophil count and **(B)** neutrophil count in patients with stable angina (SA) or at time from onset of chest pain in patients with ST-segment elevation myocardial infarction (MI). ∗p < 0.05 relative to patients with SA. SA patients: n = 307; ST-elevation MI patients: n = 732. **(C, D)** Eosinophil peroxidase immunopositive eosinophils were located in within the infarct zone of human postmortem hearts (typical of n = 4), **(E, F)** distribution was sparse in comparison to *Gr-1* immunopositive neutrophils identified in the same samples. **(C, inset)** A negative control section stained with an IgG of the same class as the EPX antibody. eo = eosinophil; ne = neutrophil.

In 732 patients presenting with STEMI (Supplemental Table 3), blood eosinophil counts declined over the first 7 to 9 h after the onset of chest pain and were suppressed compared with patients with stable angina (Figure 1A). Peripheral blood neutrophil counts in STEMI patients remained elevated at a similar level following the onset of chest pain (Figure 1B), while there continued to be a decline in eosinophil count over the same time period. Histological staining of 4 human postmortem hearts (Figures 1C to 1E), collected from patients who died within 24 h of MI (Supplemental Table 3), revealed the presence of eosinophil peroxidase immunopositive eosinophils within the infarct area, although the distribution was sparse in comparison with neutrophils identified in the same tissue (Figures 1E and 1F).

### Eosinophils are activated following experimental MI and accumulate in the infarcted left ventricle

**Figure 2:**
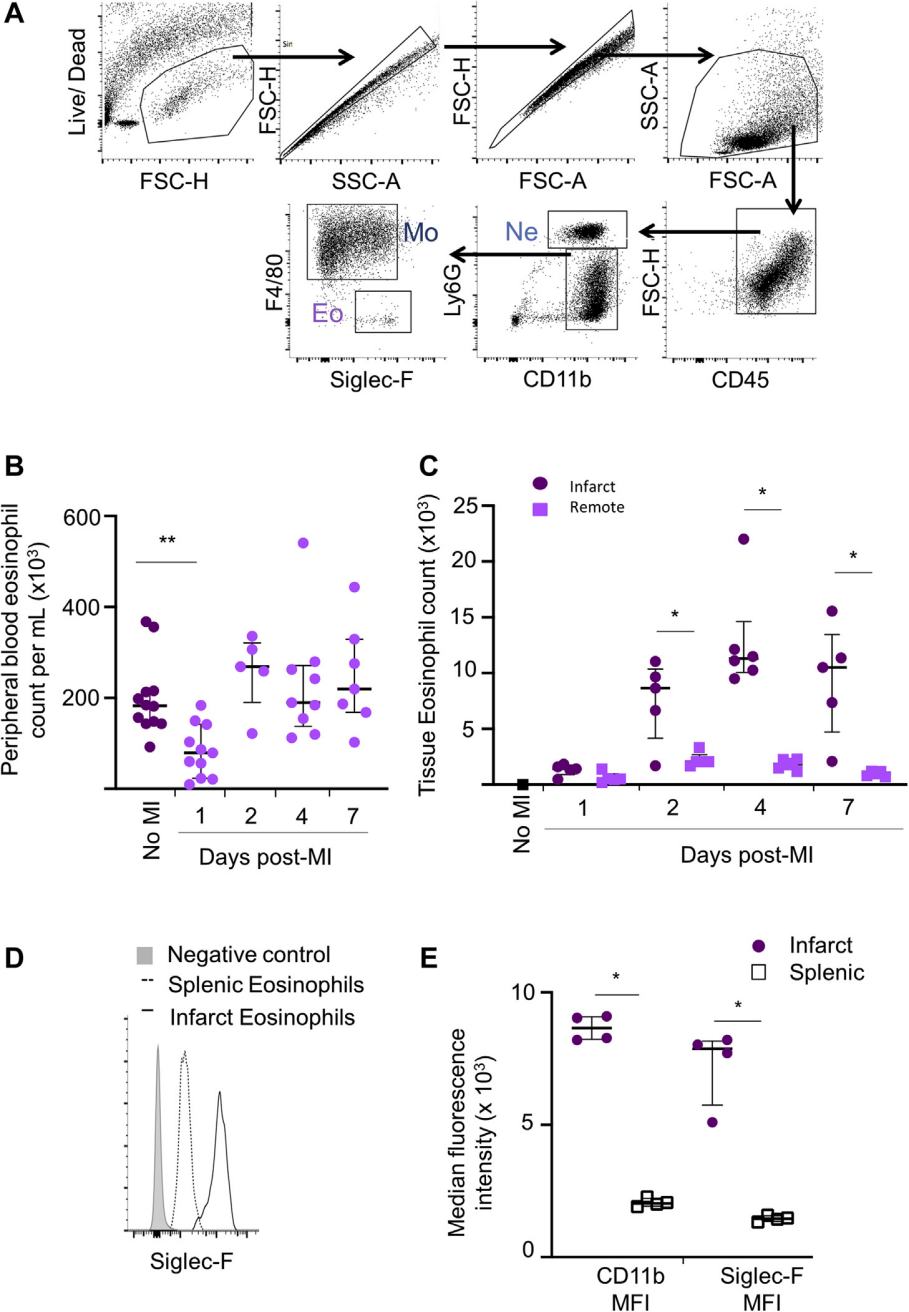
Eosinophils Are Reduced in the Blood and Accumulate in the Heart Following Experimental Myocardial Infarction in Mice **(A)** Representative flow cytometry plots showing the gating strategy applied to the left ventricle of wild-type BALB/c mice for detection of neutrophils (Ne), macrophages (Mo), and eosinophils (Eo). **(B)** Peripheral blood eosinophil count in BALB/c mice following MI (no MI: n = 12; other time points: n = 5 to 11 per group). **(C)** Total number of Siglec-F^+^Ly6G^int^ eosinophils detected by flow cytometry in the infarct and remote zones following MI (no MI: n = 3; other time points: n = 5 to 6 per group). **(D)** Representative histogram showing Siglec-F staining of splenic and infarct zone eosinophils at day 4 post-MI. **(E)** CD11b and Siglec-F median fluorescence intensity (MFI) of infarct and splenic eosinophils at day 4 post-MI (n = 4 per group). Median values with 25th and 75th percentiles are shown. ∗p < 0.05, ∗∗p < 0.01. Eo = eosinophil; MI = myocardial infarction; Mo = macrophage; Ne = neutrophil.

**Figure 3:**
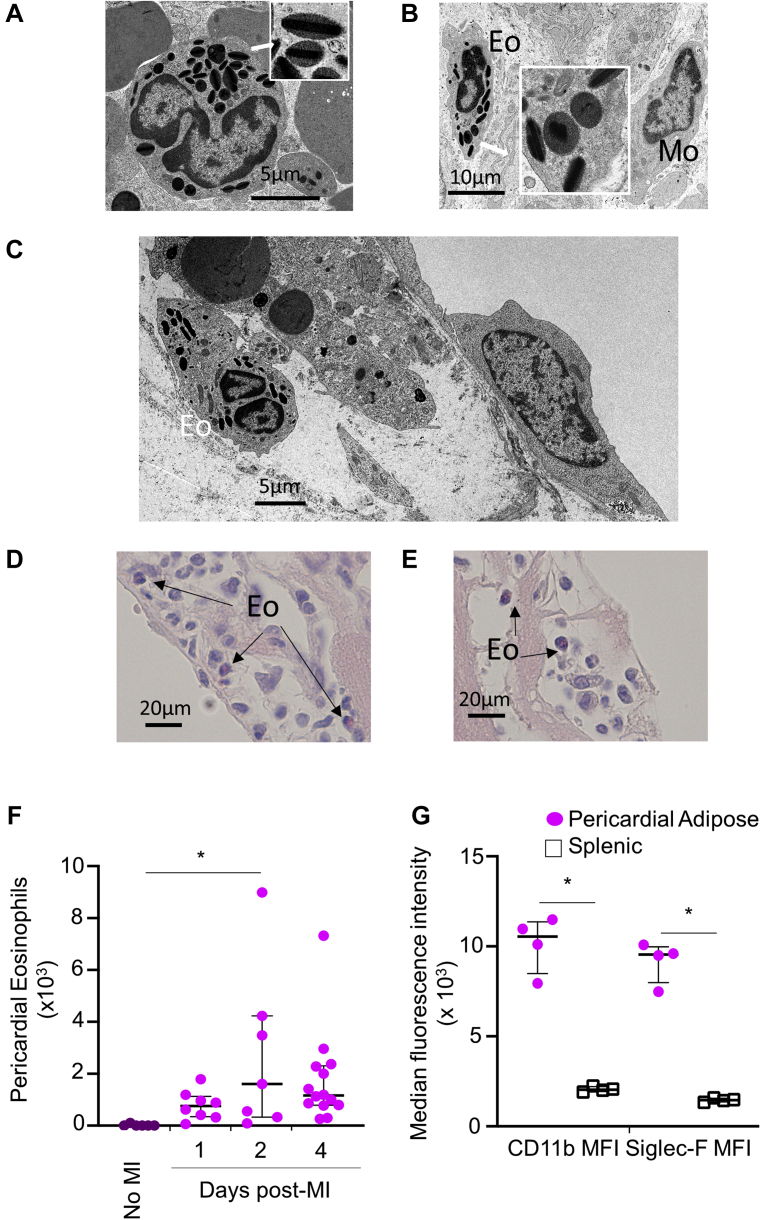
Eosinophils Locate to the Heart Following Experimental MI in Mice, With Particular Accumulation Close to the Epicardium Transmission electron microscopy (TEM) reveals **(A)** quiescent eosinophils in spleen, with typical electron dense crystalloid containing granules **(inset)** and **(B)** an eosinophil adjacent to a macrophage in infarct 4 days post-MI, varied granule morphology is consistent with eosinophil activation **(inset)**. **(C)** TEM showing an eosinophil close to the epicardial border within the infarct. Chromotrope R staining of fixed sections show eosinophils located **(D)** close to the epicardial border and **(E)** in the pericardial adipose tissue. **(F)** Flow cytometry of pericardial adipose tissue collected at 4 days post-MI shows accumulation of eosinophils relative to pericardial adipose from uninfarcted heart (no MI). CD11b and Siglec-F MFI of pericardial adipose and splenic eosinophils at day 4 post-MI (n = 4 per group). Median values with 25th and 75th percentiles are shown. ∗p < 0.05. Abbreviations as in Figure 2.

To permit further interrogation of the role of eosinophils in the heart following MI, eosinophil numbers in the blood and heart were also investigated in an experimental mouse model of MI following coronary artery ligation. Flow cytometric evaluation of Siglec-F^+^ eosinophils in blood and single-cell digests of the left ventricle (Figure 2A) revealed that, in parallel with clinical observations, there was a peripheral blood eosinopenia after experimental MI (p = 0.005) (Figure 2B). In keeping with previous studies (12), Siglec-F^+^Ly6G^int^ eosinophils were rarely found in uninjured hearts (Figure 2C). However, eosinophils were recruited to the heart from day 1 post-MI, and particularly to the infarct zone, where their numbers peaked at day 4 post-MI, during infarct repair (Figure 2C). Activation of recruited eosinophils was confirmed by flow cytometry that showed a higher intensity of Siglec-F expression on cells infiltrating the inflamed myocardium relative to naïve eosinophils residing in splenic lymphoid tissue (Figures 2D and 2E) (13). The presence of eosinophils in tissue at day 4 was confirmed by their characteristic morphology on electron microscopy, with crystalloid containing granules in the spleen (Figure 3A) and in the infarcted heart (Figure 3B). In the infarct, variable granule morphology (Figure 3B, inset) is consistent with eosinophil activation, as suggested by flow cytometry. At 4 days after MI, eosinophil numbers peaked at 2% of CD11b positive cells in the infarct, and as in the human tissue samples (Figure 1), distribution in the infarct was relatively sparse. Interestingly, electron microscopy (Figure 3C) and chromotrope R staining (Figures 3D and 3E) revealed that eosinophils could often be identified in the epicardial area. Flow cytometry also showed accumulation of Siglec-F–expressing activated eosinophils (Figures 3F and 3G) in the adjacent pericardial adipose tissue, suggesting that as a potential route of entry or activity.

### Infarct expansion and detrimental post-MI remodeling are enhanced following genetic depletion of eosinophils

**Figure 4:**
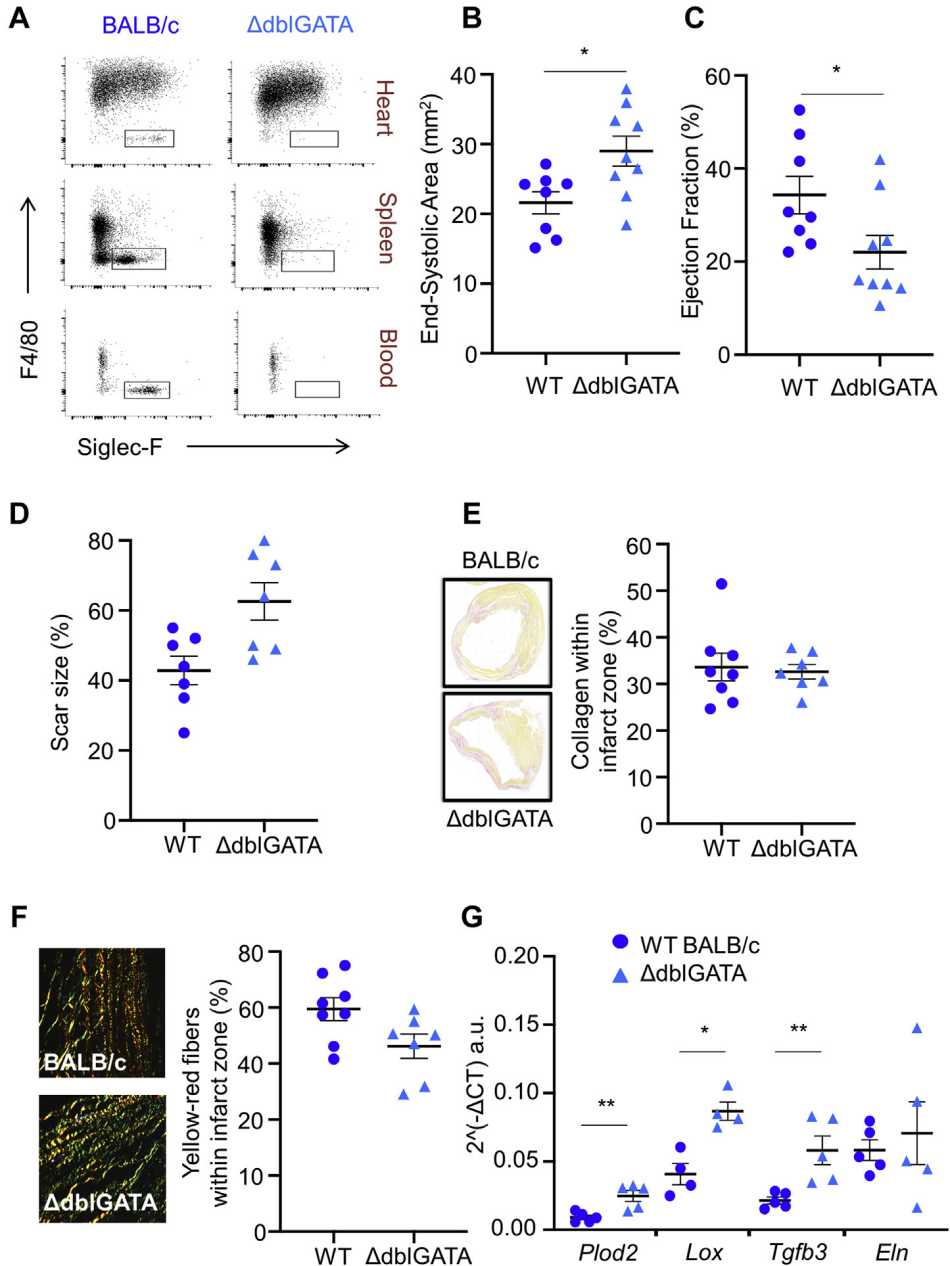
Genetic Depletion of Eosinophils Results in Enhanced Detrimental Remodeling Post–Myocardial Infarction Accompanied by Alteration of Collagen Scar Size, Morphology, and Collagen Processing Genes **(A)** Representative flow cytometry plots demonstrating (Siglec-F^+^Ly6G^int^F4/80^–^) eosinophil deficiency in the heart, spleen, and blood of ΔdblGATA mice in comparison with BALB/c mice. **(B)** End-systolic area and **(C)** ejection fraction at day 7 following MI in wild-type (WT) BALB/c and ΔdblGATA mice (n = 8 to 9 per group). **(D)** Masson’s trichrome–stained sections for assessment of scar size, expressed as a percentage of the left ventricle. **(E)** Picrosirius red–stained sections for assessment of the proportion of collagen in the infarct zone. **(F)** Imaging of picrosirius red–stained collagen fibers in the infarct zone under polarized light reveals a reduced proportion of collagen fibers in the infarct zone with yellow-red birefringence (n = 7 to 8 per group), the remaining fibers had green birefringence. **(G)** Messenger RNA expression levels of genes expressing collagen processing genes procollagen-lysine, 2-oxoglutarate 5-dioxygenase 2 (*Plod2*) and lysyl oxidase (*Lox*); transforming growth factor beta 3 (*Tgfb3*) that regulates *Plod2* expression; and elastin (*Eln*) in the infarct zone at day 7 following MI (n = 4 to 5 per group). ∗p < 0.05; ∗∗p < 0.01.

To investigate the role of eosinophils recruited to the heart following injury, MI was induced in ΔdblGATA mice with genetic deficiency of eosinophils (8). In keeping with previous findings (8), analysis of peripheral blood showed no significant differences between WT BALB/c and ΔdblGATA mice with respect to white blood cell counts at baseline (Supplemental Figure 1). Deficiency of Siglec-F^+^Ly6G^int^ eosinophils in the infarct and remote zones of the left ventricle, as well as in the spleen, of ΔdblGATA mice was confirmed by flow cytometry (Figure 4A). Left ventricular function and geometry were similar in ΔdblGATA and WT BALB/c mice prior to induction of MI (Supplemental Table 4). Following the induction of MI, high-resolution ultrasound showed that hearts from ΔdblGATA mice were more dilated (increased left ventricular area; p = 0.021) (Figure 4B, Supplemental Table 5) and had greater impairment of left ventricular function than did WT BALB/c mice (left ventricular ejection fraction; p = 0.038) (Figure 4C). Plasma troponin I concentration at 24 h post-MI was comparable between ΔdblGATA (25.7 ± 4.7 ng/ml) and WT BALB/c mice (27.0 ± 3.6 ng/ml; p = 0.832), indicating similar initial myocardial injury. However, by day 7 following induction of MI, scar size was larger in ΔdblGATA mice (Figure 4D). There was no influence of eosinophil depletion on the extent of angiogenesis post-MI (Supplemental Figure 2) or on the proportion of collagen in the infarct (Figure 4E). However, picrosirius red staining under polarizing light revealed that the proportion of tightly packed collagen fibers in the infarct zone, which have yellowish-red birefringence, was reduced in ΔdblGATA mice (Figure 4F). This was associated with an increased preponderance of thin collagen fibers, which appear green under polarized light, in the infarct zone of ΔdblGATA mice (Figure 4F). Infarct zone whole tissue qPCR at day 7 (Figure 4G) for genes associated with post-translational collagen processing showed increased mRNA expression of procollagen-lysine, 2-oxoglutarate 5-dioxygenase 2 (*plod2*) (p = 0.009), lysyl hydroxylase 2, lysyl oxidase (*Lox*) (p = 0.029), and transforming growth factor beta 3 (*tgfb3*) (p = 0.008) in ΔdblGATA mice in comparison with WT BALB/c mice. mRNA expression of elastin (*Eln*) in the infarct zone of ΔdblGATA mice was comparable to that in WT BALB/c mice. These findings support a role for eosinophils in collagen scar maturation following MI.

### Pharmacological intervention confirms enhancement of detrimental remodeling following eosinophil depletion

**Figure 5:**
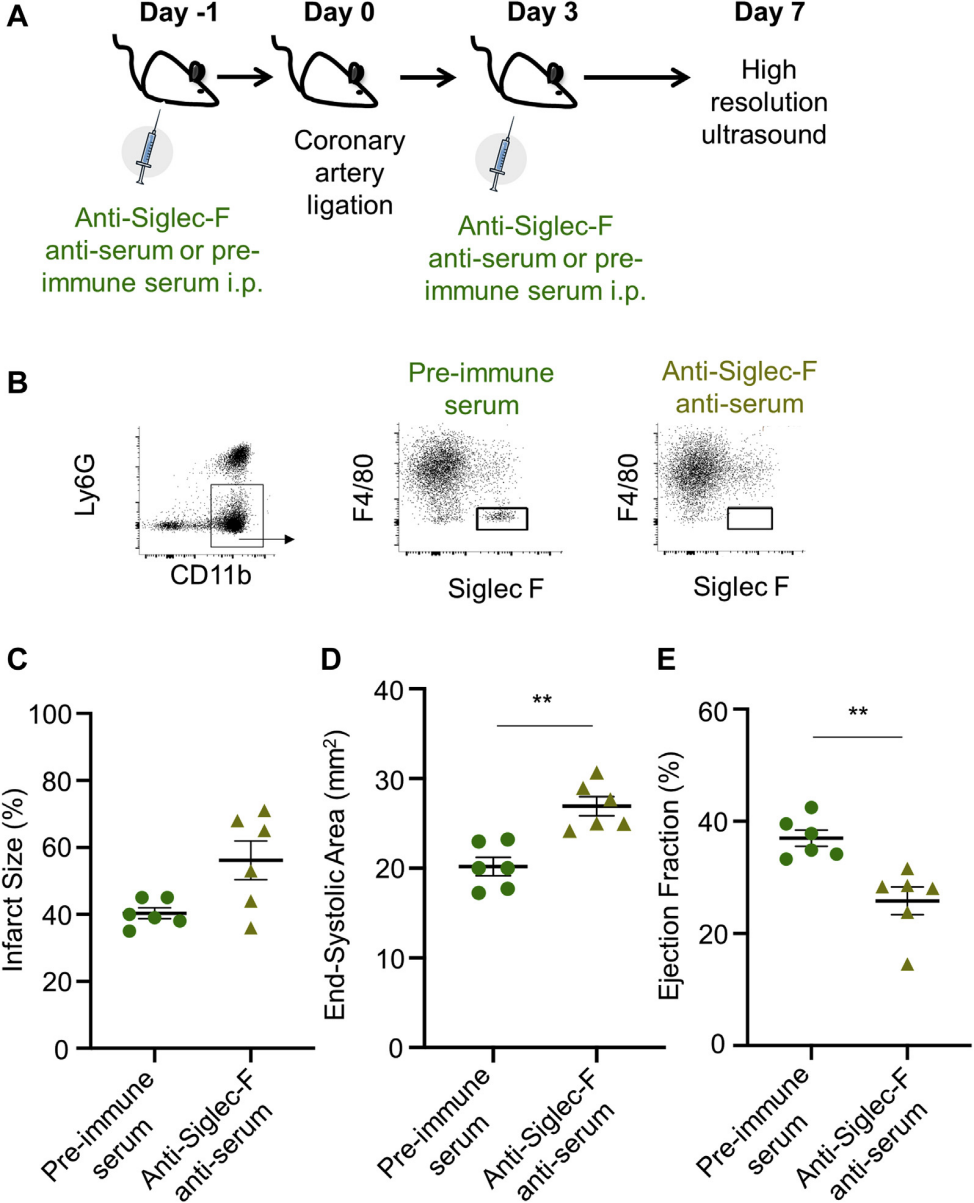
Pharmacologic Depletion of Eosinophils Reproduces Adverse Remodeling in C57BL/6 Mice **(A)** C57BL/6 mice were treated with anti-Siglec-F anti-serum 1 day before and 3 days after induction of myocardial infarction (MI). **(B)** Flow cytometry reveals successful depletion of Siglec-F^+^Ly6G^int^ eosinophils within the infarcted heart following antiserum treatment. **(C)** Scar size, **(D)** end-systolic area, and **(E)** ejection fraction at day 7 following MI in pre-immune serum–treated (n = 6) and anti-Siglec-F anti-serum–treated C57BL/6 mice (n = 6). ∗p < 0.05, ∗∗p < 0.01. i.p. = intraperitoneal.

To confirm the data obtained with genetic deficiency of eosinophils in BALB/c mice, an antibody-mediated approach was used as an alternative means of depleting eosinophils following MI in C57BL/6 mice (Figure 5A). Successful depletion of Siglec-F^+^Ly6G^int^ eosinophils using anti-Siglec-F antiserum was confirmed by flow cytometry of single-cell digests of infarcted hearts at day 4 post-MI (Figure 5B). Depletion of eosinophils did not affect initial injury induced by MI, as measured by plasma troponin I concentrations at 24 h post-MI (Supplemental Table 6). By 7 days after MI, scar size in tissues tended to be increased in anti-Siglec-F antiserum–treated mice (Figure 5C) (p = 0.074), and this was accompanied by adverse remodeling of the left ventricle. High-resolution ultrasound showed that hearts from anti-Siglec-F antiserum–treated mice were more dilated (Figure 5D) and had greater impairment of left ventricular function than did pre-immune serum–treated mice (Figure 5E and Supplemental Table 6).

Thus, pharmacological depletion of eosinophils in C57BL/6 mice, that have a distinct inflammation and repair phenotype compared with BALB/c mice (14), confirmed the adverse cardiac remodeling phenotype associated with eosinophil depletion, further supporting a role for eosinophils in post-MI remodeling.

### Eosinophils support an anti-inflammatory environment in the infarct zone

**Figure 6:**
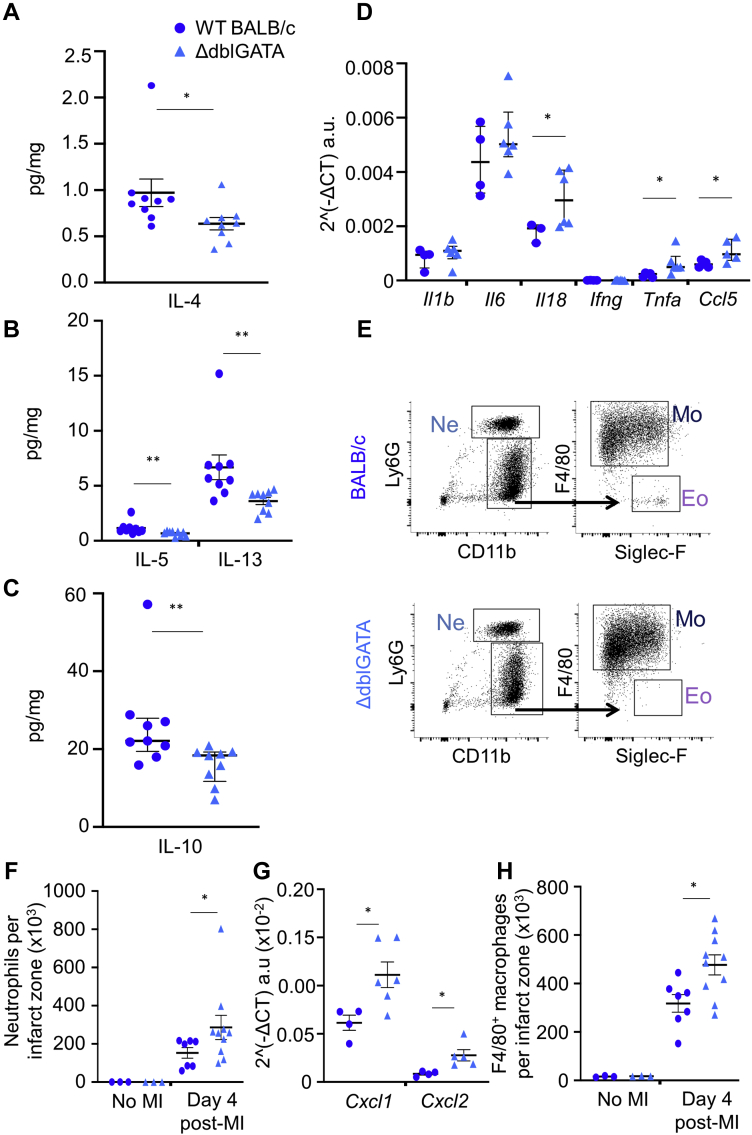
Eosinophils Are Required for Acquisition of an Anti-Inflammatory Th2-Dominant Environment During Infarct Repair. Enzyme-Linked Immunosorbent Assay Reveals Reduced Availability of Th2 Cytokines **(A)** Interleukin (IL)-4, **(B)** IL-5 and IL-13, and **(C)** IL-10 in the infarct zones of ΔdblGATA compared with wild-type (WT) mice at day 4 following MI (n = 9 per group; median values with 25th and 75th percentiles are shown); and **(D)** increased messenger RNA (mRNA) expression of proinflammatory IL-1β (Il1b), IL-6 (Il6), IL-18 (Il18), interferon-γ (Ifng), tumor necrosis factor-α (Tnfa), chemokine (C-C motif) ligand 5 (*Ccl5*) in the infarct zone at day 7 following MI (n = 4 to 6 per group; median values with 25th and 75th percentiles are shown). mRNA expression levels were normalized for Rpl32 (housekeeping gene) expression. **(E)** Representative flow cytometry plots showing the gating strategy applied to the infarct zone tissue of wild-type (WT) BALB/c and ΔdblGATA mice for detection of neutrophils, macrophages, and eosinophils. **(F)** Total number of neutrophils in the uninfarcted heart (no-MI) and in the infarct zone 4 days after MI. **(G)** mRNA expression levels of chemokine (C-X-C motif) ligand 1 (*Cxcl1*) and chemokine (C-X-C motif) ligand 2 (*Cxcl2*) in the infarct zone following MI (n = 4 to 6 per group). mRNA expression levels were normalized for Rpl32 (housekeeping gene) expression. **(H)** Total number of F4/80^+^ macrophages in the uninfarcted heart (no MI) and in the infarct zone 4 days after MI (no MI: n = 3; day 4 post-MI: n = 7 to 10 per group). ∗p < 0.05, ∗∗p < 0.01. Abbreviations as in Figure 2.

Anti-inflammatory Th2-type cytokines have a key role in the resolution of inflammation and are elevated in the infarct zone at day 4 post-MI (15). As eosinophils are known to support a type 2 immune environment during tissue repair and regeneration, we next aimed to investigate the influence of eosinophil depletion on type 2 immune mediator availability in the infarct. In keeping with an anti-inflammatory role for eosinophils, enzyme-linked immunosorbent assay showed that the infarct tissue of ΔdblGATA mice had significantly reduced availability of IL-4, IL-5, IL-13, and IL-10 (Figures 6A to 6C), in comparison with WT BALB/c mice at day 4 following MI. In contrast, expression of genes encoding the proinflammatory mediators IL-18 (*Il18*), chemokine (C-C motif) ligand 5 (*Ccl5*), and tumor necrosis factor-α (*Tnfa*) were all elevated in the infarct zone of ΔdblGATA mice compared with WT BALB/c mice at day 7 post-MI (Figure 6D). Characterization of myocardial inflammatory cell content using flow cytometry (Figure 6E) showed that the number of infarct zone Ly6G^hi^Ly6C^int^ neutrophils was higher in ΔdblGATA mice at day 4 post-MI, relative to WT BALB/c mice (Figure 6F) (p = 0.025). Infarct zone whole-tissue qPCR for genes associated with neutrophil recruitment showed increased mRNA expression of both chemokine (C-X-C motif) ligand 1 (*Cxcl1*) (p = 0.038) and chemokine (C-X-C motif) ligand 2 (*Cxcl2*) (p = 0.016) (Figure 6H). Infarct zone F4/80^+^ macrophage numbers were also increased in ΔdblGATA mice (Figure 6H) (p = 0.019) following MI. Together, these data demonstrate impaired resolution of inflammation in the absence of eosinophils.

### Delayed acquisition of a prorepair macrophage phenotype in eosinophil deficient myocardium is rescued by eosinophil replacement

**Figure 7:**
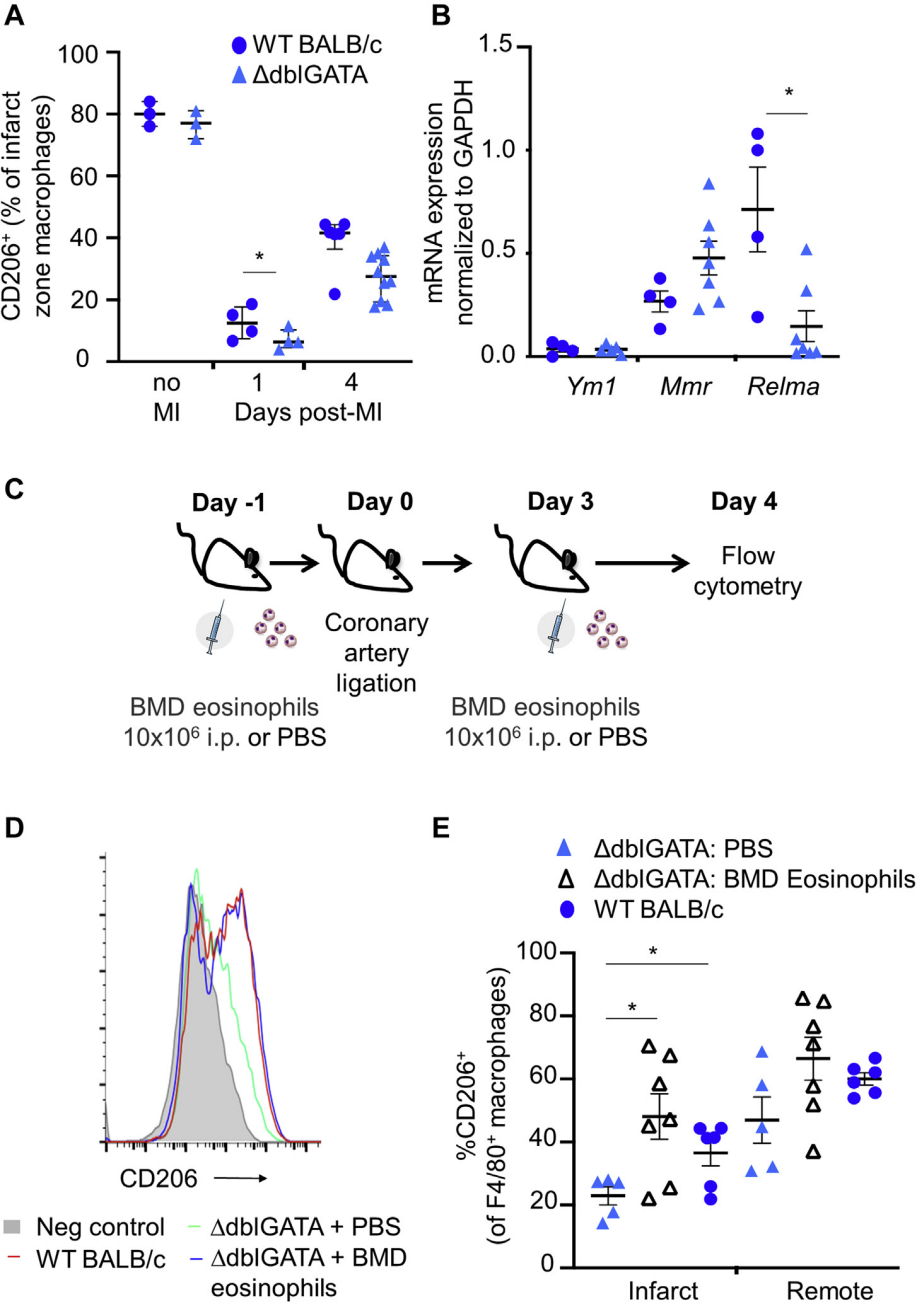
Delayed Acquisition of a Prorepair Macrophage Phenotype in Eosinophil-Deficient Myocardium Is Rescued by Eosinophil Replacement **(A)** The proportion of CD206-expressing F4/80^+^ macrophages in unoperated hearts (no myocardial infarction [MI]) and in the infarct zone at days 1 and 4 post-MI in ΔdblGATA compared with wild-type (WT) BALB/c mice (no MI: n = 3; other time points: n = 4 to 10 per group; median values with 25th and 75th percentiles are shown). **(B)** Messenger RNA (mRNA) expression levels of chitinase-like 3 (*Ym1*), macrophage mannose receptor (*Mmr*), and resistin-like molecule α (*Relma*) in macrophages sorted from the infarct zone at day 7 following MI (n = 4 to 8 per group). **(C)** Protocol for delivery of bone marrow–derived (BMD) eosinophils, or phosphate-buffered saline (PBS), 1 day before and 3 days after MI. **(D)** Representative histogram of CD206-expressing F4/80^+^ macrophages in the infarct zones of PBS-treated or BMD eosinophil–replenished ΔdblGATA mice at day 4 post-MI. **(E)** Proportion of CD206-expressing F4/80^+^ macrophages in the infarct and remote zones of PBS-treated or BMD eosinophil–replenished ΔdblGATA mice at day 4 post-MI (n = 5 to 7 per group). ∗p < 0.05. i.p. = intraperitoneal.

Resolution of inflammation following MI occurs with a transition toward an anti-inflammatory macrophage phenotype that is essential for repair and prevention of adverse cardiac remodeling (2). CD206 expression is typically increased on these alternatively activated anti-inflammatory macrophages (2). The next aim was therefore to investigate whether eosinophils were required for acquisition of a CD206^+^ anti-inflammatory macrophage phenotype following MI. CD206 was present on nearly 80% of macrophages in naïve hearts (Figure 7A). Within the first 24 h after induction of MI, representation of CD206^+^ macrophages was reduced to <20% and F4/80^+^CD206^-^ proinflammatory macrophages dominated in the infarct zone (Figure 7A). On day 4 post-MI, nearly 40% of infarct zone macrophages from WT BALB/c once again expressed CD206 (Figure 7A). However, acquisition of CD206 expression by infarct zone macrophages was impaired at day 4 post-MI in ΔdblGATA mice (Figure 7A), supporting key roles for eosinophils in driving infarct zone macrophage polarization toward an anti-inflammatory phenotype following MI. Expression of the mRNA encoding the anti-inflammatory macrophage marker, resistin-like molecule α (*Retnla/*RELMα), was also reduced in macrophages sorted from the infarct zones of ΔdblGATA (p = 0.024) compared with BALB/c mice (Figure 7B) (n = 4 to 8 per group).

To confirm that loss of macrophage polarization during infarct repair was due to eosinophil deficiency, BMD eosinophils were adoptively transferred by intraperitoneal injection into ΔdblGATA mice before and after MI (Figure 7C). Adoptive transfer of BMD eosinophils resulted in restoration of CD206 expression on infarct zone macrophages from ΔdblGATA mice to levels of those from WT BALB/c mice (Figures 7D and 7E).

### Adverse cardiac remodeling associated with eosinophil deficiency is rescued by therapeutic IL-4 complex

**Figure 8:**
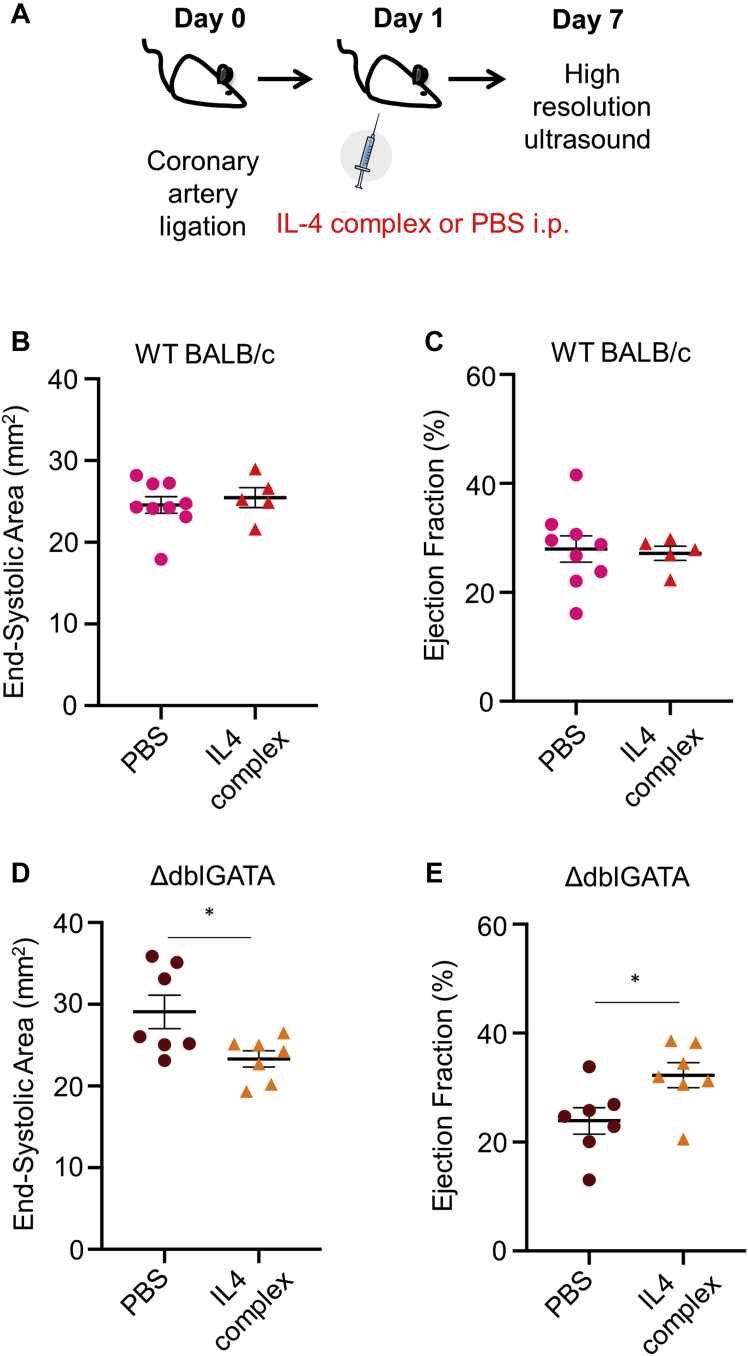
IL-4 Therapy Rescues the Excess Adverse Remodeling Phenotype in Eosinophil-Deficient ΔdblGATA Mice **(A)** Protocol for administration of IL-4 complex 1 day after induction of MI. **(B)** End-systolic area and **(C)** ejection fraction at day 7 following MI in WT BALB/c mice injected i.p. with PBS or IL-4 complex (n = 5 to 9 per group). **(D)** End-systolic area and **(E)** ejection fraction at day 7 following MI (n = 7 per group) in ΔdblGATA mice injected i.p. with PBS or IL-4 complex. ∗p < 0.05; ∗∗p < 0.01. Abbreviations as in Figure 7.

As type 2 cytokine availability is reduced in the setting of eosinophil deficiency in ΔdblGATA mice, it was reasoned that therapeutic application of IL-4 might be an effective means to rescue adverse remodeling. A long-acting IL-4 complex was therefore administered to ΔdblGATA and WT BALB/c mice 24 h after induction of MI (Figure 8A). Plasma troponin I concentrations at 24 h post-MI were comparable between WT BALB/c and ΔdblGATA mice prior to treatment with either PBS or IL-4 complex (Supplemental Tables 7 and 8), indicating similar initial injury in all groups. High-resolution ultrasound showed that while IL-4 complex had no impact on remodeling in eosinophil-replete WT BALB/c mice when given 24 h after injury (Figures 8B and 8C and Supplemental Table 7), it was able to rescue the adverse remodeling phenotype in eosinophil-deficient ΔdblGATA mice (Figures 8D and 8E and Supplemental Table 8).

## Discussion

Blood eosinophil count is reduced in the acute period following MI, and we have previously shown that low peripheral blood eosinophil count predicts adverse outcome (5). Analysis of human myocardium collected postmortem following MI identified eosinophils, suggesting that they are recruited to the site of injury, consistent with previous identification of eosinophils in postmortem myocardium collected following MI (16). However, distribution was relatively sparse in the infarct, and this clinical observation leaves open the question of whether eosinophil recruitment to the injured heart has any negative or positive influence on subsequent repair and remodeling. Eosinophil recruitment has previously been linked to promotion of detrimental remodeling in chronic heart failure (17). In addition, it cannot be excluded that death may have influenced eosinophil recruitment to the infarct zone in postmortem samples. To address these questions, the role of eosinophils was investigated further using an experimental model of MI in the mouse that permits manipulation of eosinophil availability.

### Eosinophils are recruited to the heart and activated following experimental MI

In the mouse model, eosinophil numbers in the peripheral blood also declined after MI, and this was associated with accumulation of eosinophils in the myocardium that began within 24 h and continued to increase to a peak at 4 days after MI. The temporal mismatch between the decline of eosinophil numbers in the blood and accumulation in the tissue indicates a more complex regulation of peripheral blood eosinophils than can be accounted for simply by recruitment to tissue. Inflammatory cytokines produced by activated innate immune cells (18) and stress responses, mediated by adrenal corticosteroids (19), can lead to eosinophil apoptosis and contribute to a decline in peripheral blood eosinophil count. Clarifying how the inflammatory environment following MI influences eosinophil survival during the period of eosinophil recruitment to the infarct zone will in the future provide clues to better understand the association of peripheral blood eosinophil count with outcome in MI patients (5, 6, 7).

Recruitment of eosinophils to the repairing mouse myocardium, reproducing the clinical observation, is consistent with an active role for eosinophils following MI. This is further supported by the observation that on becoming resident in the infarcted heart, alteration of granule morphology (4,13) is accompanied by increased expression of Siglec-F, indicating a switch from a homeostatic to an activated phenotype (13,20). Overall, eosinophil recruitment appeared to be low in the heart relative to other CD11b^+^ cells, suggesting that interaction with neighboring cells may be required to amplify their influence in infarct repair. Identification of eosinophils in the pericardial adipose and near to the epicardium is interesting, particularly given the recent identification of key roles for cells at these sites in determination of inflammation, scar formation, and cardiac function after MI (21, 22, 23, 24). The potential for interactions of eosinophils at these sites merits further investigation.

### Eosinophil depletion results in adverse remodeling associated with impaired scar formation

A functional role for eosinophils was confirmed in vivo in eosinophil-deficient ΔdblGATA BALB/c mice (8), and also in C57BL/6 mice with antibody-mediated eosinophil depletion with anti-Siglec-F antiserum (13). Both models of eosinophil depletion were accompanied by an increase in adverse structural and functional remodeling, indicating that, despite their low representation relative to neutrophil and macrophages, eosinophils have a key positive impact on myocardial repair and remodeling following their recruitment from the blood. Importantly, this outcome was clear in mice that are more (BALB/c) or less (C57BL/6) skewed toward Th2 immune dominance (14), showing that the influence of eosinophils is not dependent on mouse strain or immune phenotype. Adverse remodeling was accompanied by an increase in scar size in the myocardium of ΔdblGATA mice relative to WT, despite no difference in the initial ischemia-induced injury. Angiogenesis was unchanged in eosinophil-deficient mice, suggesting that infarct expansion did not occur due to reduced blood supply in the infarct border. Formation of a stable collagen scar is key for determination of wall stress, infarct expansion, and subsequent ventricular remodeling following MI. There was no indication that the amount of collagen, revealed by picrosirius red staining, was different between samples. However, investigation of the infarct scar using birefringence microscopy revealed modification of collagen fibril formation in eosinophil-deficient mice. Eosinophil depletion also resulted in increased infarct zone expression of *plod2*, encoding lysyl hydroxylase 2 (LH2), and also *tgfb*3, encoding transforming growth factor beta 3 (TGF-β3), which upregulates *plod2* expression in fibroblasts (25). Increased *plod2* expression leads to increased post-translational collagen crosslinking, resulting in reduced tensile strength (26, 27, 28). The detrimental effects of increased collagen crosslinking have been identified in the weakened aortic wall present in Marfan syndrome and abdominal aortic aneurysms (28). Therefore, disrupted post-translational collagen processing in the infarct zone may underlie infarct expansion and ventricular dilatation observed following MI in the absence of eosinophils. Evidence is now accumulating that epicardial fibroblasts have a key role in the formation of infarct scar (21,23). Identification of eosinophils in the epicardium presents the intriguing possibility that they might influence fibroblast phenotype either directly or indirectly, via interaction with other cells, at this location.

### Eosinophils are required to promote resolution of inflammation during post-infarct repair

Inflammation is essential for infarct repair but when excessive or prolonged is associated with adverse cardiac remodeling (29). In other settings, eosinophil-derived proresolving lipid mediators can reduce the numbers of neutrophils in inflamed tissue (3,4) by counter-regulating neutrophil influx and stimulating macrophage phagocytosis of apoptotic neutrophils (5), thus promoting the resolution of inflammation. Neutrophil recruitment to the heart following MI was increased in eosinophil-deficient ΔdblGATA mice, and this was associated with increased expression of proinflammatory mediators, including neutrophil-recruiting chemokines. Eosinophil-deficient ΔdblGATA mice also had reduced availability of the anti-inflammatory Th2-type cytokines, IL-4, IL-5, IL-13, and IL-10, in the infarct tissue. This was accompanied by a reduction in the proportion of infarct zone macrophages expressing CD206^+^ and RELM-α, that identify anti-inflammatory, proresolution, and prorepair macrophage phenotypes in the infarct zone (2). Following MI, CD206^+^ macrophages produce paracrine mediators that activate cardiac fibroblasts promoting scar formation (2). Consistent with our data, Qin et al. (30) have recently demonstrated increased expression of CD206 and RELM-α when cultured macrophages were treated with human eosinophil–conditioned media. In the current study, loss of CD206^+^ macrophages in the infarct of eosinophil-deficient ΔdblGATA mice was rescued by eosinophil replenishment. Recent single-cell sequencing studies have provided new evidence for the complexity of macrophage phenotypes found in the myocardium (31), with varied potential roles in determining resolution of inflammation, repair, and remodeling (32). Collectively, the findings presented here support a role for eosinophils in driving transition to resolution of inflammation and stable scar formation during infarct repair. Further studies will be required to identify the impact of eosinophil depletion on the distribution of specific macrophage phenotypes.

### Therapeutic IL-4 rescues adverse post-MI in the setting of eosinophil deficiency

IL-4–mediated activation of macrophages is known to induce characteristic expression of specific effector molecules (e.g., RELM-α) and is associated with the adoption of a wound healing phenotype (33). Previous experiments have demonstrated the importance of this phenotype during post-MI repair (2) and the ability of therapeutic IL-4 to enhance accumulation of CD206^+^ “wound-healing” macrophages and prevent detrimental remodeling, at least when administered immediately after injury (3). Eosinophils are a key source of IL-4, and indeed other molecules, in noncardiac tissue repair and regeneration (20,34,35). Experiments in eosinophil-deficient mice showed that IL-4 availability in the infarct was significantly reduced at day 4 following MI, the time point at which eosinophil recruitment to the myocardium peaks, and also a key time point for transition of macrophages from an inflammatory to a repair phenotype (36). Given the relative sparsity of recruited eosinophils in the heart after MI, it seems unlikely that they are the primary source of IL-4 here; however, they might influence the formation of IL-4 and other cytokines by neighboring cells in the infarct milieu. Interestingly, administration of IL-4 complex 24 h after MI, when eosinophil recruitment to the myocardium is already significantly increased, had no influence on post-MI remodeling in eosinophil-replete mice. This is consistent with the previous studies that showed therapeutic benefit of IL-4 only when given prior to (2) or immediately after (3) MI. However, IL-4 complex administration was effective in reversing adverse cardiac remodeling in eosinophil-deficient mice. This positive outcome could result from direct replacement of IL-4 missing in eosinophil-deficient mice or perhaps from activation by therapeutic IL-4 of proresolution and prorepair mechanisms that are able to overcome the combined effects of eosinophil depletion.

In summary, this study identifies eosinophils as key effector cells of the type 2 innate immune response required for acute post-MI cardiac repair. IL-4 therapy, already proposed as a means to improve outcomes post-MI (3), was effective in reversing detrimental outcomes in the setting of eosinophil deficiency in experimental MI. The use of biomarkers such as a persistently low peripheral blood eosinophil count post-MI may therefore provide a means to direct IL-4 therapy to patients who might gain the most benefit from it.

Perspectives **COMPETENCY IN MEDICAL KNOWLEDGE:** Formation of a stable scar and resolution of inflammation are central features of successful infarct repair following myocardial infarction. Eosinophils appear to have a key role in these processes.

**TRANSLATIONAL OUTLOOK:** Further studies are needed to evaluate prospectively the prognostic predictive value of a low peripheral blood eosinophil count and the potential therapeutic efficacy of IL-4 in preventing adverse cardiac remodeling in patients following myocardial infarction.
