# Supplementary material for: Eosinophil Deficiency Promotes Aberrant Repair and Adverse Remodeling Following Acute Myocardial Infarction
Source: JACC Basic Transl Sci. 2020 Jul 8;5(7):665–81. doi: 10.1016/j.jacbts.2020.05.005 (PMC7393409; doi:10.1016/j.jacbts.2020.05.005)
Supplement: Supplemental Tables 1–9 and Supplemental Figures 1 and 2 [file mmc1.pdf]

SUPPLEMENTAL MATERIAL

SUPPLEMENTAL FIGURE 1 Peripheral Blood Cell Characterization

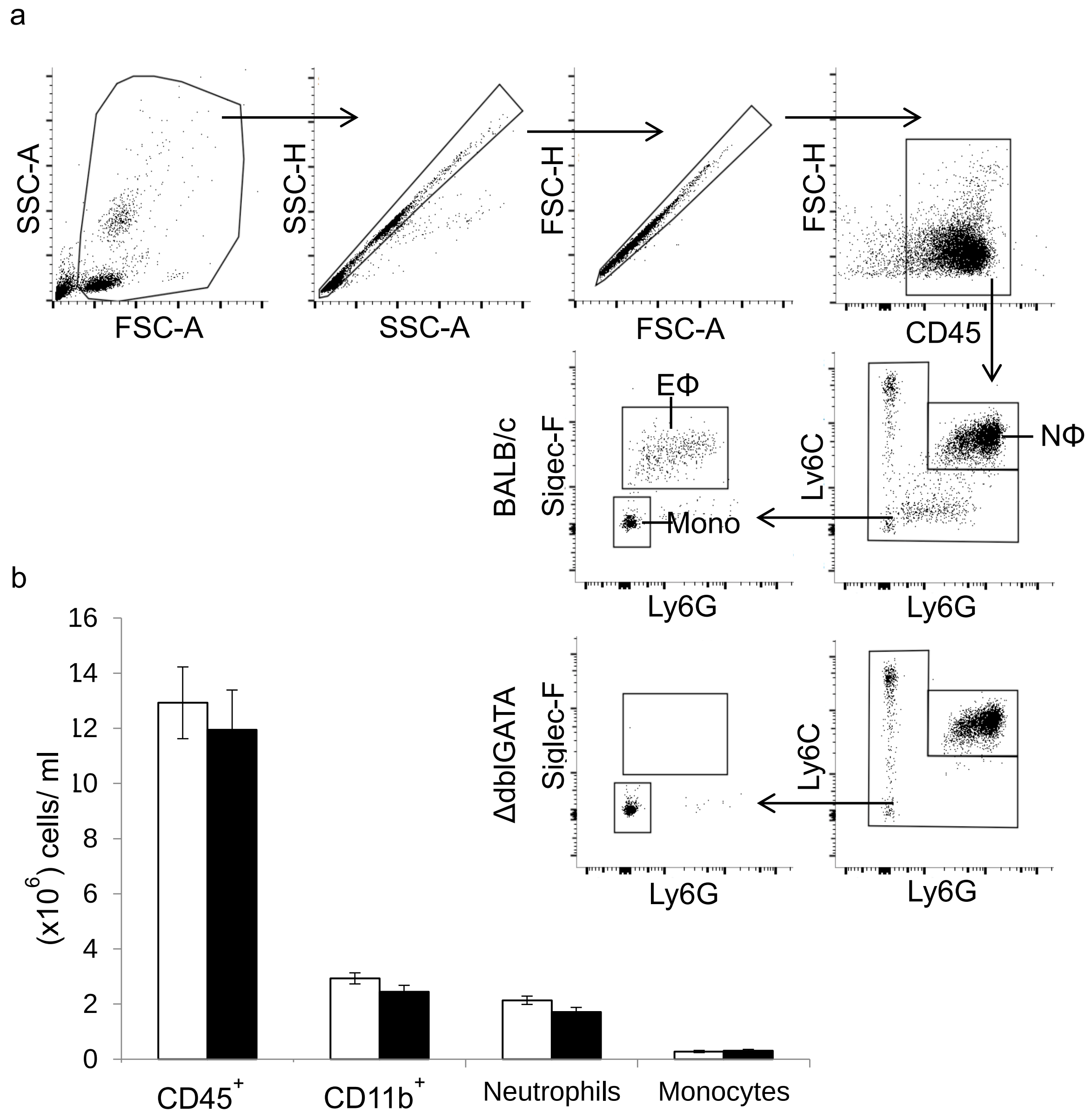

(a) Representative flow cytometry plots showing the gating strategy applied to the peripheral blood of WT BALB/c and ΔdblGATA mice. **(b)** Baseline peripheral blood white blood cell counts in Wild-Type BALB/c and ΔdblGATA mice. White bars: Wild Type BALB/c mice n=9. Black bars: ΔdblGATA mice n=5. Data re resented as mean ± SEM.

SUPPLEMENTAL FIGURE 2 Characterization of post-infarct neovascularization in wild-type (WT Balb C) and eosinophil deficient (ΔdblGATA) mice.

(a)

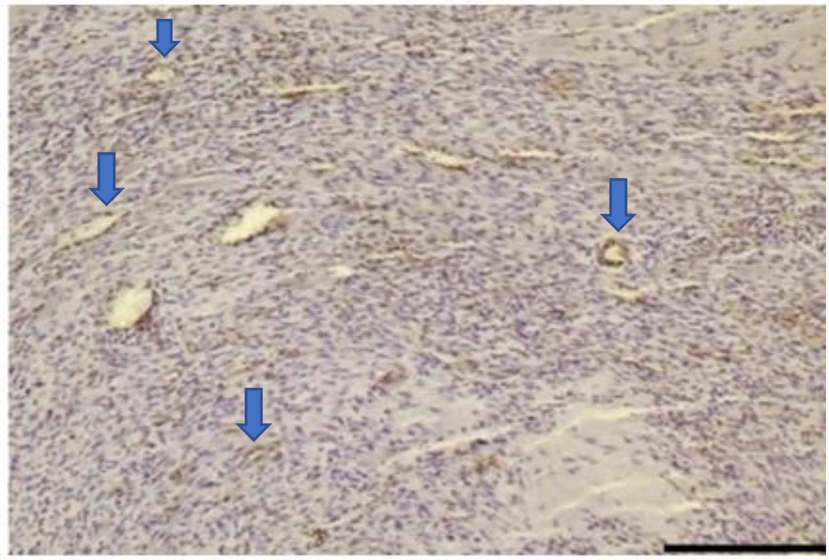

(b)

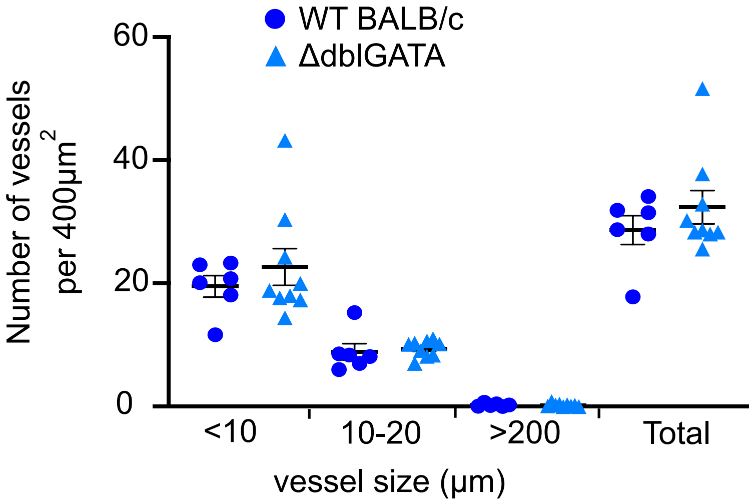

(a) CD31 immunopositive blood vessels in the infarct at 7 days after MI. Scale bar is 100µm, section is counterstained with H&E. (b) Quantification of $CD31^{+ve}$ vessels of varying size in the infarct zone expressed per $400µm^2$ (n=6-9 /group).

SUPPLEMENTAL TABLE 1. **Clinical Data and Autopsy Results for Donors of Post-MI Myocardial Tissue Used in Immunohistochemistry**

	**Clinical Data**	**Autopsy Results**
**Patient 1**	71-year-old male<br>Time elapsed since infarction: 24 hours<br>Cause of death: myocardial infarction	Acute luminal thrombosis of the right coronary artery. Myocyte contraction band necrosis with an acute interstitial inflammatory cell infiltrate of the posterior left ventricle.
**Patient 2**	72-year-old male<br>Time elapsed since infarction: 24 hours<br>Cause of death: myocardial infarction	Focal severe atheroma in the left anterior descending coronary artery. Confluent myocyte contraction band necrosis in the anterior left ventricle, with an associated acute interstitial inflammatory cell infiltrate.
**Patient 3**	52-year-old male<br>Time elapsed since infarction: 24 hours<br>Cause of death: myocardial infarction	Focal acute luminal thrombosis of coronary artery. Foci of myocardial contraction band necrosis with acute inflammatory cells within the interstitium.
**Patient 4**	46 year old male<br>Time elapsed since infarction: 48h<br>Cause of death: myocardial infarction	Triple vessel coronary artery disease, thrombus in left anterior descending coronary artery. Transmural infarction, myocyte contraction band necrosis, neutrophil infiltrate of left ventricle.

MI = myocardial infarction.

SUPPLEMENTAL TABLE 2: **Primer IDList**

Gene	Gene name	Assay ID
Chemokine (C-C motif) ligand 5	Ccl5	Mm01302427_m1
Chitinase-like 3	Chil3	Mm00657889_mH
Collagen type Iα1	Col1a1	Mm00801666_g1

Collagen type IIIα1	Col3a1	Mm00802300_m1
Elastin	Eln	Mm00514670_m1
Interleukin-1β	Il1b	Mm00434228_m1
Interleukin-6	Il6	Mm00446190_m1
Interleukin-18	Il18	Mm00434226_m1
Interferon-γ	Ifng	Mm01168134_m1
Macrophage mannose receptor	Mmr	Mm00443781_m1
Matrix metalloproteinase-2	Mmp2	Mm00439498_m1
Procollagen-lysine,2-oxoglutarate 5-dioxygenase 2	Plod2	Mm00478767_m1
Resistin-like molecule α	Retnla	Mm00445109_m1
Ribosomal protein L32	Rpl32	Mm02528467_g1
Tissue inhibitors of matrix metalloprotease 3	Timp3	Mm00441826_m1
Transforming growth factor, beta 3	Tgfb3	Mm00436960_m1
Tumor necrosis factor-α	Tnfa	Mm00443258_m1

SUPPLEMENTAL TABLE 3: Baseline characteristics of stable angina and STEMI patients

	Stable Angina patients N=307	STEMI patients N=732	p-value
Age (yrs)	65.8 ± 0.5	64.6 ± 0.5	0.372
Male	237 (77%)	547 (75%)	0.434
Diabetes Mellitus	79 (26%)	87 (12%)	<0.001
Hypertension	189 (62%)	282 (39%)	<0.001
History of smoking	153 (50%)	374 (51%)	0.634
Hypercholeste-rolaemia	250 (81%)	182 (25%)	<0.001
CVA	10 (3%)	36 (5%)	0.317
Previous MI	96 (31%)	92 (13%)	<0.001
Previous CABG	26 (67%)	13 (33%)	<0.001
Previous PCI	113 (65%)	61 (35%)	<0.001
PVD	8 (3%)	14 (2%)	0.483
Admission Creatinine (mmol/L)	90 ± 1	87 ± 1	0.252

CVA = cerebrovascular accident, CABG = coronary artery bypass surgery, MI = myocardial infarction, PCI = percutaneous coronary intervention, PVD = peripheral vascular disease, STEMI = ST-segment Elevation Myocardial Infarction. Data are presented as mean± SEM, the $\chi 2$ tests was used for comparison of categorical variables.

SUPPLEMENTAL TABLE 4. **Cardiac Function at Baseline in Wild-Type Balb/C Mice and ΔdblGATA Mice**

	BALB/c (n = 6)	ΔdblGATA (n = 5)	p Value
Heart rate, beats/min	467 ± 12	456 ± 17	0.626
End-diastolic area, mm^2	27.0 ± 0.9	26.3 ± 0.8	0.566
End-systolic area, mm^2	18.9 ± 0.6	17.2 ± 0.9	0.382
Ejection fraction, %	46.4 ± 1.0	54.4 ± 2.7	0.284
LVIDs (mm)	3.0 ± 0.1	3.2 ± 0.3	0.533
LVPWs (mm)	0.9 ± 0.03	1.0 ± 0.1	0.379
FS (%)	25 ± 2	26 ± 2	0.886

Value are mean ± SEM.

FS = fractional shortening; LVIDs = left ventricular internal diameter at end-systole; LVPWs = left ventricular posterior wall thickness at end systole.

SUPPLEMENTAL TABLE 5. **Cardiac Injury (24h post-MI), Cardiac Structure and Heart Rate at Day 7 Following MI in Balb/C and ΔdblGATA Mice**

	BALB/c (n = 8)	ΔdblGATA (n = 9)	p Value
24h troponin I, ng/ml	27.0 ± 3.6	25.7 ± 4.7	0.832
Heart rate, beats/min	471 ± 14	465 ± 7	0.700
End-diastolic area, mm^2	27.1 ± 1.1	33.4 ± 1.7	0.009
LVIDs (mm)	3.2 ± 0.2	4.6 ± 0.5	0.024
LVPWs (mm)	1.2 ± 0.1	1.1 ± 0.1	0.601
FS (%)	25.1 ± 3.7	12.4 ± 3.4	0.032

Value are mean ± SEM.

FS = fractional shortening; LVIDs = left ventricular internal diameter at end-systole; LVPWs = left ventricular posterior wall thickness at end systole.

SUPPLEMENTAL TABLE 6. Cardiac Injury (24h post-MI), Cardiac Function and Heart Rate at Day 7 Following MI in C57Bl/6 Mice Injected Intraperitoneally with Pre-Immune Serum or Anti-Siglec-F Anti-Serum on 1 Day Before and Day 3 Post-MI

	Pre-Immune Serum (n = 6)	Anti-Siglec-F Anti-Serum (n = 6)	p Value
24h troponin I, ng/ml	21.9 ± 4.2	20.9 ± 3.2	0.491
Heart rate, beats/min	552 ± 13	569 ± 5	0.244
End-diastolic area, mm^2	26.9 ± 1.4	31.9 ± 1.0	0.009
LVIDs (mm)	3.0 ± 0.2	3.9 ± 0.2	0.015
LVPWs (mm)	1.1 ± 0.1	1.1 ± 0.1	0.599
FS (%)	28.4 ± 2.0	18.9 ± 2.5	0.009

Value are mean ± SEM.

FS = fractional shortening; LVIDs = left ventricular internal diameter at end-systole; LVPWs = left ventricular posterior wall thickness at end systole.

SUPPLEMENTAL TABLE 7. **Cardiac Injury (24h post-MI), Cardiac Function and Heart Rate at Day 7 Following MI in Balb/C Mice Injected Intraperitoneally with PBS or IL-4 Complex Containing 5 mg rIL-4 on Day 1 Post-Mi**

	PBS (n = 9)	IL-4 Complex (n = 5)	p Value
24h Troponin I, ng/ml	26.4 ± 3.6	30.2 ± 4.4	0.520
Heart rate, beats/min	478 ± 14	496 ± 22	0.474
End-diastolic area, mm^2	29.4 ± 0.9	30.2 ± 1.4	0.616
LVIDs (mm)	3.5 ± 0.2	3.2 ± 0.1	0.438
LVPWs (mm)	1.2 ± 0.1	1.5 ± 0.1	0.083
FS (%)	22.1 ± 3.4	27.5 ± 1.9	0.438

Value are mean ± SEM.

IL-4 = interleukin-4; PBS = phosphate-buffered saline; rIL-4 = recombinant IL-4. FS = fractional shortening; LVIDs = left ventricular internal diameter at end-systole; LVPWs = left ventricular posterior wall thickness at end systole.

SUPPLEMENTAL TABLE 8. **Cardiac Injury (24h post-MI), Cardiac Function and Heart Rate at Day 7 Following MI in ΔdblGATA Mice Injected Intraperitoneally with PBS or IL-4 Complex Containing 5mg rIL-4 on Day 1 Post-MI**

	PBS (n = 7)	IL-4 complex (n = 7)	p Value
24h troponin I, ng/ml	25.9 ± 4.5	20.8 ± 2.9	0.329
Heart rate, beats/min	462 ± 12	482 ± 20	0.402
End-diastolic area, mm^2	32.9 ± 2.4	28.9 ± 1.0	0.154
LVIDs (mm)	4.6 ± 0.3	3.7 ± 0.2	0.026
LVPWs (mm)	0.8 ± 0.1	1.1 ± 0.09	0.050
FS (%)	12.8 ± 2.1	21.3 ± 3.0	0.039

Value are mean ± SEM.

IL-4 = interleukin-4; PBS = phosphate-buffered saline; rIL-4 = recombinant IL-4. FS = fractional shortening; LVIDs = left ventricular internal diameter at end-systole; LVPWs = left ventricular posterior wall thickness at end systole.

SUPPLEMENTAL TABLE 9. **Antibodies for Flow Cytometry**

Monoclonal Rat Anti-Mouse ab	Clone	Fluorophores	Manufacture	Concentration
CD45.2	104	PE Cy7	BioLegend	1:100
CD45.2	104	BV650	BioLegend	1:500
CD11b	M1/70	AF700	BioLegend	1:200
F4/80	BM8	PE Cy7	BD Biosciences	1:200
Ly6G	1A8	Pacific Blue	BioLegend	1:200
Siglec-F	E50-2440	AF647	BD Pharmingen	1:100
Siglec-F	E50-2440	PE	BD Pharmingen	1:100
CD206	C068C2	FITC	BioLegend	1:100
Ly6C	HK1-4	PerCP Cy5.5	BD Pharmingen	1:100
CD115	T38-320	APC	BD Pharmingen	1:100
CD115	T38-320	DAPI	Life Technologies	1:1000
Anti-CD16/32	2.4G2	—	BD Biosciences	1:200
